# Validation of digit-length ratio (2D:4D) assessments on the basis of DXA-derived hand scans

**DOI:** 10.1186/s12880-015-0042-7

**Published:** 2015-02-03

**Authors:** Michael Romann, Jörg Fuchslocher

**Affiliations:** Swiss Federal Institute of Sport Magglingen, CH-2532 Magglingen, Switzerland

**Keywords:** Talent identification, Young athletes, Finger length, Measurement techniques

## Abstract

**Background:**

The second-to-fourth digit-length ratio (2D:4D) may be a correlate of prenatal sex steroids, and it has been linked to sporting prowess. The aim of the study was to validate dual-energy X-ray-absorptiometry (DXA) as a technique to assess 2D:4D in soccer players under 15 years of age (U-15).

**Methods:**

Paired X-ray and DXA scans of the left hands of 63 male U-15 elite soccer players (age: 14.0 ± 0.3 years) were performed, and 2D:4D was then compared between the two techniques. The 2D:4D measurements were performed twice by two blinded raters. Intrarater and interrater reliability, as well as agreement between the X-ray and the DXA assessments, were tested.

**Results:**

Intrarater reliabilities of both raters using X-ray with intraclass correlation coefficients (ICCs) of 0.97 and 0.90 were excellent. Using DXA, the ICCs were 0.90 and 0.91 thus also showing excellent reliability. Interrater reliabilities were excellent using both the X-ray (ICC of 0.94) and the DXA (ICC of 0.90), assessments respectively. Bland-Altman plots demonstrated that the 2D:4D ratios of the two raters did not differ significantly between the X-ray and the DXA assessments. The standard errors of estimate were 0.01 for both techniques. The 95% limits of agreement of ±0.018 (±2.0%) and ±0.023 (±2.6%), respectively, were within the acceptable tolerance of 5%, and showed very good agreement.

**Conclusion:**

DXA offered a replicable technique for assessing 2D:4D in youth soccer players. Therefore, the DXA technique seems to be an alternative method for evaluating 2D:4D in youth sports.

## Background

The ratio of the second and fourth digits (2D:4D) seems to correlate with the amount of prenatal testosterone [1]. 2D:4D is sexually dimorphic in humans such that males show lower mean values of 2D:4D than females. Sexual dimorphism appears early in the human fetus [2] and stays stable during childhood and adolescence [3]. Several methods have been used to assess 2D:4D. These techniques include direct measurements with calipers [4], inked handprints [5] measurements from photocopies [6], scanned images [7], digital photographs [8], scaled tubes [9], self-reporting online [10], and X-ray measurement [11,12]. Each of these methods has limitations relating to its feasibility and costs; however, in general, direct measurements, photocopies, and scanned images are the most commonly used techniques [13].

In comparison to these methods, radiographic measurements deliver the most accurate measurement of bone length, and X-ray measurements are more accurate and precise than direct or photographic methods [14]. However, the main problem with using X-ray imaging with children and adolescents is the exposure to radiation [15-17]. In modern technology, the assessment of 2D:4D by hand radiography requires 1 μSv of radiation, which is the equivalent of less than four hours of natural background radiation or 10 minutes on an intercontinental flight [18,19]. Nevertheless, to avoid the possible detrimental effects of cumulative radiation exposure, children and adolescents should only be exposed to a minimal amount of radiation [15,20]. Consequently, when assessing 2D:4D reducing the radiation dose is an important issue, and methods involving less radiation are preferable, particularly in children and adolescents. Dual-energy X-ray absorptiometry (DXA) is the most commonly used bone densitometric technique used for children, worldwide [21]. The main advantages of this technique are its low cost, high precision, speed, availability, and safety [21]. DXA-derived hand scans [22] have recently become available. Evaluating 2D:4D via hand radiographs using DXA produces a 10-fold lower effective dose (0.1 μSv) than using X-ray (1 μSv), with results that appear to be comparable to those of standard radiographs [21,23]. Therefore, using DXA to assess 2D:4D could be a possible approach for evaluating 2D:4D.

Researchers have highlighted a considerable range of variables that are predictors for future success in the development and selection of athletic talent. There are obvious variables, such as an athlete’s strength and performance and the quantity and quality of training [24], or psychological factors, such as motivation [25]. Other variables, including the athlete’s date of birth [26,27], the role of older siblings [28], and 2D:4D [1] are less obvious. In this manner 2D:4D has been identified as a biomarker for performance in various elite sports [1]. A smaller ratio is believed to be an indicator of greater testosterone exposure, which may lead to superior sports performance [6,29].To the best of our knowledge, no study has investigated the agreement between DXA and X-ray hand imaging as techniques for assessing 2D:4D. As 2D:4D has been linked to athletic prowess, agreement between 2D:4D calculations using X-ray and DXA as imaging techniques should be analyzed. Until now, the applicability of DXA scans for assessing 2D:4D in youth athletes has not been validated where 2D:4D might play an important role. Therefore, the aim of the present study was to validate DXA as a technique for assessing 2D:4D of soccer players under 15 years of age.

## Methods

Participants were recruited from among the 72 soccer players who were invited to the Swiss Soccer Association’s national selection day. One of this study’s authors (the project leader) invited all 72 players to participate in the study. The cross-sectional 2D:4D sample included 63 (87.5%) male soccer players who decided to participate in the study. All of the participants were in good health and free of acute or known chronic diseases at the time of the study. The study was approved by the local research ethics committees (Kantonale Ethikkommission Bern, Switzerland, No. 022/13). Written informed consent was obtained from all of the parents and each of the participants. The participants were informed that participation was voluntary and that they could withdraw from the study at any time. Weight, height, and 2D:4D were measured. To measure weight, the participants wore shorts and a T-shirt. Height was measured with a stadiometer (Seca 217; Seca, Hamburg, Germany), and weight was measured with calibrated scales (Tanita WB-110 MA; Tanita, Tokyo, Japan). Weight and height were measured to the nearest 0.1 kg and 0.1 cm, respectively. All of the hand-wrist X-rays and DXA scans were performed at the Swiss Olympic Medical Center Magglingen according to hand-wrist guidelines. With the participants sitting beside the X-ray device (Stadler SE 4600; Stadler, Littau, Switzerland), the left hand-wrist was placed without any radial or ulnar deviance on a double-layered phosphor cassette. Using this standardization, posterior-anterior radiographs of the left hand were taken with an X-ray device. All digital X-ray scans were analysed on a computer screen using iQ-VIEW 2.5.0 (IMAGE Information Systems, London, UK). A standardized modus of 42 kV tube voltage and 1.60 mAs, with a radiation time of 0.78 s, was used. Subsequently, on the same day, each participant underwent a DXA scan of the left hand-wrist (iDXA; General Electric Lunar, 2008, Madison, WI). Scans were analysed using software supplied by GE Medical Systems (GE enCORE 2011, version 13.60). This software allows a left hand wrist scan which were performed with all participants. Automatic brightness and contrast optimisation was used. All the scans were executed by one investigator using a standardized modus of 100 kV tube voltage and 0.19 mAs. With the participants sitting beside the DXA device, the scan was focused on the hand-wrist, starting four centimeters below the radiocarpal joint in order to obtain an image of the distal radius, the wrist, and all of the hand bones. All of the X-ray and DXA images were saved without any participant characteristics in order to insure that all of the analyses were blind assessments. All of the scans were analyzed by two independent, trained raters (R1, R2). The measurements were made with precision clear rulers to the nearest of 0.5 mm. The distances of 2D and 4D were measured from the base of the proximal phalanx to the tip of the distal phalanx [30]. R1 and R2 independently assessed each of the participants’ 2D:4Ds on the X-ray and the DXA scan to evaluate interrater variability, and they analyzed these a second time four weeks later to evaluate intrarater reliability. The assessments were repeated after four weeks in order to minimize recall bias. Residuals were examined for normality, linearity. The normality assumption was checked using Kolmogorov-Smirnov (KS) test and linearity with lack of fit test. To compare the two techniques employed to assess 2D:4D, we used the statistical methods described by Bland and Altman [31]. Intrarater and interrater reliability were analyzed using intraclass correlation coefficients (ICCs) with a 95% confidence interval (CI). Values of less than 0.40 indicated poor reliability, values of 0.40-0.60 indicated fair reliability, values of 0.60-0.75 indicated good reliability, and values greater than 0.75 indicated excellent reliability [32]. Bland-Altman plots were used to visualize the differences between the X-rays and the DXA scans and their distribution. The mean 2D:4D, the mean difference, the standard deviation (SD) of the mean difference, 95% limits of agreement (LoA), and standard error of the estimate (SEE) were calculated. In accordance with previous validation studies, we decided to accept that the mean difference between the two techniques could deviate by a maximum of 5% from the mean of both techniques and to accept that the LoA could be within a range of ±0.05. Statistical analysis was performed using the Statistical Package for the Social Sciences (SPSS) version 21.0 (IBM SPSS, Chicago, Illinois, USA). The level of significance was set at P < 0.05.

## Results

The mean age of the participants was 14.0 ± 0.3 years, mean height was 164.9 ± 8.4 cm, and mean weight was 53.0 ± 8.7 kg. Data of finger length and differences between X-ray and DXA assessments were normally distributed (KS, p > 0.05) and linearity was given (lack of fit, p > 0.05). Table 1 shows the intrarater reliability of the 2D:4D calculations using X-rays and DXA scans. For R1, the intrarater difference between the two assessments using X-rays was 0.001 with an SEE of 0.005 and an ICC of 0.97 (0.94–0.98). Using DXA, the difference was 0.001 with an SEE of 0.009 and an ICC of 0.90 (0.85–0.93). For R2, the intrarater difference between the two assessments using X-rays was 0.001 with an SEE of 0.010 and an ICC of 0.91 (0.85–0.95). Using DXA, the difference was 0.001 with an SEE of 0.009 and an ICC of 0.91 (0.85–0.94). According to the classifications of Rosner [32] the intrarater reliabilities of both raters were excellent.

**Table 1:** **Intrarater reliability of 2D:4D measurements**

**Observer**	**Method**	**mean 2D:4D (SD)**	**∆**	**TEM (%)**	**SEE**	**ICC (95% CI)**	**Classification**
Observer 1	RX	0.905 (0.020)	0.001	0.004 (0.6%)	0.005	0.97 (0.94-0.98)	excellent
	DXA	0.908 (0.019)	0.001	0.006 (0.8%)	0.009	0.90 (0.85-0.93)	excellent
Observer 2	RX	0.904 (0.020)	0.001	0.006 (0.8%)	0.010	0.91 (0.85-0.95)	excellent
	DXA	0.907 (0.020)	0.001	0.007 (0.9%)	0.009	0.91 (0.85-0.94)	excellent

Note: Intrarater reliability (repeatability) for both raters. = difference; TEM = technical error of measurement, absolut and in % of the mean. SEE = standard error of estimate; ICC = intraclass correlation coefficients; CI = confidence intervall; classification according to Rosner (2011). ICCs < 0.7 were considered non-acceptable, 0.71 < ICCs < 0.79 were acceptable, 0.80 < ICCs < 0.89 were very good and ICCs >0.90 were excellent.

The mean 2D:4D interrater difference between the assessments using X-rays was 0.001, with an SEE of 0.010 and an ICC of 0.94 (0.91–0.96). The mean 2D:4D interrater difference between the assessments using DXA was 0.001, with an SEE of 0.010 and an ICC of 0.90 (0.86–0.94). The interrater reliabilities were excellent with both assessment techniques (Table 2).

**Table 2:** **Interrater reliability of 2D:4D measurements**

**Method**	**Observer 1**	**Observer 2**	**∆**	**TEM (%)**	***SEE***	**ICC (95% CI)**	**Classification**
	**mean 2D:4D (SD)**	**mean 2D:4D (SD)**					
X-ray	0.905 (0.020)	0.904 (0.020)	0.001	0.007 (0.9%)	0.010	0.94 (0.91-0.96)	excellent
DXA	0.908 (0.019)	0.907 (0.020)	0.001	0.009 (1.2%)	0.010	0.90 (0.86-0.94)	excellent

Note: Interrater reliability for both observers. = difference; TEM = technical error of measurement, absolut and in % of the mean. SEE = standard error of estimate; ICC = intraclass correlation coefficients; CI = confidence intervall; classification according to Rosner (2011). ICCs < 0.7 were considered non-acceptable, 0.71 < ICCs < 0.79 were acceptable, 0.80 < ICCs < 0.89 were very good and ICCs >0.90 were excellent.

**Figure 1:**
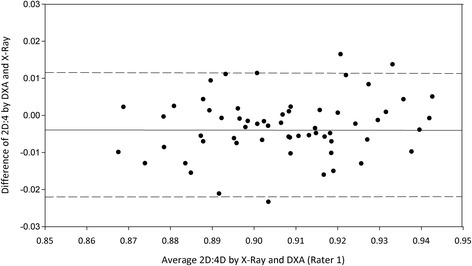
**Bland-Altman plot of the variation between DXA and X-ray assessments of rater 1.** The continuous line indicates mean and the two dashed lines indicate the 95% limits of agreement.

**Figure 2:**
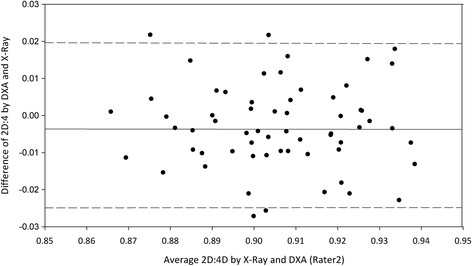
**Bland-Altman plot of the variation between DXA and X-ray assessments of rater 2.** The continuous line indicates mean and the two dashed lines indicate the 95% limits of agreement.

Agreement between the assessments is shown in the Bland-Altman plots. For each participant, the difference between the X-ray and DXA assessments is plotted against the mean of these assessments (Figures 1 and 2). The LoAs (mean ± 1.96 SD) are plotted in both figures. The differences between the X-ray and DXA assessments were normally distributed. The mean difference between R1’s 2D:4D measurements was 0.003 (0.4%), with an SEE of 0.010 and an ICC of 0.89 (0.83-0.93) (Table 3). The 95% LoA was ± 0.018 (±2.0%). The mean difference between R2’s measurements was 0.003 (0.4%), with an SEE of 0.010 and an ICC of 0.81 (0.74-0.87). The 95% LoA was ± 0.023 ((±2.6%). All paired assessment points lie close to the horizontal line of mean differences, indicating good agreement and suggesting small differences between the techniques. Moreover, all of the points seem to lie randomly around the line of mean difference, indicating an absence of systematic bias. Correlation between the difference and means of measurements was 0.02 (p > 0.5) for rater 1 and 0.04 (p > 0.5) for rater 2.

**Table 3:** **Agreement of 2D:4D measurements**

**Observer**	**RX mean (SD)**	**DXA mean (SD)**	**∆**	**TEM (%)**	**SEE**	**ICC (95% CI)**	**Classification**
Observer 1	0.905 (0.020)	0.908 (0.019)	0.003	0.009 (1.2%)	0.010	0.89 (0.83-0.93)	very good
Observer 2	0.904 (0.020)	0.907 (0.020)	0.003	0.011 (1.4%)	0.010	0.81 (0.74-0.87)	very good

Note: Intrarater reliability (repeatability) for both raters. = difference; TEM = technical error of measurement, absolut and in % of the mean . SEE = standard error of estimate; ICC = intraclass correlation coefficients; CI = confidence intervall; classification according to Rosner (2011). ICCs < 0.7 were considered non-acceptable, 0.71 < ICCs < 0.79 were acceptable, 0.80 < ICCs < 0.89 were very good and ICCs >0.90 were excellent.

## Discussion

The main finding of this study was excellent agreement between DXA and X-ray derived 2D:4D assessments. The mean difference between the assessments did not deviate more than 5% and the 95% LoAs were in the range of the defined −0.05 and +0.05 limit for the 2D:4D ratio. According to these levels of agreement, the 2D:4D assessments using the DXA technique produces results that are similar to the results produced by the common assessments using the X-ray technique.

In the last decade a large body of knowledge has grown in 2D:4D research with more than 60 publications per year [33]. Specifically the relationship between 2D:4D and athletic prowess has high interest and relevance among sports science. Significant correlations between performance and 2D:4D have been shown in e.g. running, soccer, rugby, skiing, fencing and swimming [6,34-36]. There is evidence that 2D:4D is a correlate of prenatal testosterone and ghrelin concentrations in sport [6,34]. As a result 2D:4D is suggested to be a predictor for important abilities in sport like strength and aerobic capacities [37,38].

In addition to the importance and high relevance of 2D:4D research in sport the results of our study were in line with 2D:4D values found in studies performed by Manning and Hill [37] and Manning and Taylor [6]. The intrarater and interrater variances of 2D:4D using techniques other than DXA showed similar results as well (e.g., [13,39,40]). Allaway [13] assessed the level of intrarater and interrater reliability when evaluating 2D:4D using four different techniques: direct finger length measurements, photocopies, printed scanned images, and computer-assisted image analysis. In that study, the ICCs of intrarater reliability were 0.96 by computer-assisted analysis, 0.94 by photocopies, 0.93 by direct measurements and 0.84 by printed scans. The ICCs of interrater reliability were 0.89 by computer-assisted, 0.86 by photocopies, 0.80 by direct measurements, and 0.76 by printed scans. Ranson, Taylor, and Stratton [39] showed an excellent interrater ICCs reliability of 0.95 (0.92–0.97) and a very good to excellent interrater ICCs reliability of 0.90 (0.83–0.94) using photographs as an imaging technique. A longitudinal study with Jamaican children found interrater ICCs of 0.951 (left hand) and 0.940 (right hand), respectively, and four years later, ICCs of 0.977 (left hand) and 0.971 (right hand), respectively, were reported using direct measurements [3]. Peeters and Claessens [40] used X-ray measurements to calculate 2D:4D and they revealed excellent intrarater and interrater ICCs of 0.98. Compared to these results, the present study showed similar intrarater and interrater ICCs. However, in the previous studies, different study-designs were used and the experience of the raters varied; therefore, it is difficult to compare the studies’ findings.

To the best of our knowledge there are no studies in the literature that evaluated the agreement of 2D:4D calculations from X-rays and DXA scans. Our results showed that 2D:4D scans made using the DXA technique deliver valid 2D:4D values compared to scans made using the X-ray technique. The DXA technique had excellent reliability and very good agreement in comparison to the X-ray technique. We expect our results to be valid for other populations in the sports setting. Therefore, in sports, the implementation of 2D:4D measurements made by DXA scans may provide an additional parameter in the selection and development of talent. The major advantage of the DXA technique is that it produces a 10-fold lower effective dose of radiation (1 μSv) compared to the X-ray technique (0.1 μSv) [21,23]. In terms of 2D:4D assessment, one disadvantage of the DXA technique is that the definition of the scan is worse than the definition of an X-ray scan and the DXA scanning procedure is more time consuming than an X-ray. The DXA scan lasts approximately 60s (depending on the size of the wrist-hand), which increases the probability of movement artifacts. In contrast, an X-ray examination takes less than 1 s.

## Conclusion

The results obtained from using the DXA technique are similar in accuracy to those obtained by using the X-ray technique. Therefore, DXA seems to be an acceptable alternative technique for assessing 2D:4D in elite sports. The major advantage of the DXA technique compared with the classical X-ray technique is that the DXA technique has a 10-fold lower exposure to radiation. A disadvantage of using DXA for assessing 2D:4D is that the definition of the DXA scan is worse than the definition of an X-ray, and the DXA scanning procedure is more time consuming, which increases the probability of movement artifacts.
